# Development and application of a VNAR-based detection nanobody for avian influenza virus H9N2

**DOI:** 10.3389/fimmu.2026.1735255

**Published:** 2026-03-24

**Authors:** Hongzhou Ye, Qingqing Dong, Min Qian, Yanwen Guo, Yi Chen, Wenyuan Liu, Lili Liu, Zhengbing Lyu, Chen Yuan, Xiaofeng Jiang

**Affiliations:** 1 Department of Pediatrics, The First Affiliated Hospital of Huzhou University, Huzhou, China; 2 College of Life Sciences and Medicine, Zhejiang Sci-Tech University, Zhejiang Provincial Key Laboratory of Silkworm Bioreactor and Biomedicine, Zhejiang Sci-Tech University, Hangzhou, China

**Keywords:** *Chiloscyllium plagiosum*, H9N2 subtype avian influenza virus, MDCK, prevention and treatment, VNAR

## Abstract

The H9N2 subtype avian influenza virus, as a mutant strain of low-pathogenic avian influenza (LPAI), has augmented its transmission ability and pathogenicity via genetic evolution. At present, it has incurred the most substantial economic losses to the global poultry industry, particularly in Asia where it is extensively prevalent. To tackle this challenge, it is essential to devise effective prevention and control strategies for the H9N2 subtype. Among these strategies, vaccine development and highly sensitive diagnostic techniques are the primary countermeasures. Although more than twenty different vaccines and diagnostic kits for various types of avian influenza are currently available on the market, existing detection methods lack sufficient specificity for avian influenza virus subtypes. Precise subtype differentiation still depends on polymerase chain reaction (PCR)-based methods, which necessitate specialized equipment and are rarely accessible in on-site settings such as poultry farms. Our laboratory has developed specific single-domain antibodies (VNAR) derived from sharks against the H9N2 subtype of the avian influenza virus. The recombinant anti-H9N2 VNAR demonstrates high specificity for hemagglutinin (HA) binding activity and can specifically recognize, bind to, and neutralize the H9N2 subtype avian influenza virus on the surface of Madin-Darby canine kidney (MDCK) cells. Owing to their low molecular weight, excellent stability, and enhanced tissue penetration, these specific VNAR molecules possess extremely high application potential, rendering them innovative candidate drugs for the diagnosis and prevention of H9N2 infections.

## 1 Introduction

Avian influenza virus (AIV), a type A influenza virus within the *Orthomyxoviridae* family, has wild birds as its principal natural reservoir. AIV is regarded as one of the world’s major pathogenic agents of concern, given its extensive host range and remarkable transmission capacity (1, 2). According to the antigenic disparities in hemagglutinin (HA) and neuraminidase (NA), AIV can be categorized into 18 distinct subtypes (3). Different combinations of these subtypes have given rise to diverse AIV strains, among which the H9N2 subtype is a low-pathogenicity avian influenza virus (LPAIV) (4). It often triggers large-scale outbreaks in poultry farms, resulting in decreased egg production and fertility rates, thus causing substantial economic losses (5, 6).

Since its first isolation in the United States in the 1960s, H9N2 has achieved global circulation, causing poultry diseases and substantial economic damage to the industry. China experienced its first H9N2 outbreak between November 1992 and May 1994, affecting seventeen chicken farms and two exotic bird facilities. By 1998, H9N2 had rapidly spread across China and has since become the most prevalent influenza subtype in domestic chickens (7, 8). Through continuous genetic reassortment and antigenic drift, the H9N2 subtype has demonstrated increasing pathogenicity and transmission efficiency, evolving into a major pathogen threatening global poultry production (9–12). Notably, H9N2 exhibits cross-species transmission potential, posing a latent threat to public health (13, 14). Recent studies have identified H9N2 as a genetic donor for several human influenza viruses, including the H7N9 strain that first emerged in China in 2013, which acquired internal gene segments from H9N2 (10).

The primary control measures against H9N2 subtype avian influenza virus (AIV) include surveillance and vaccination (15–18). Regular poultry monitoring helps to detect emerging strains and assess their potential risks, while the development of region-specific vaccines has become a critical strategy for prevention (19–21). However, the genetic diversity of H9N2 poses challenges to vaccine efficacy, and high production costs, along with potential unknown side effects, further complicate immunization efforts (22–27). Additionally, strict biosafety measures, such as restrictions on international cross-border poultry trade and improved hygiene management in farming environments, are essential for controlling viral spread.

Avian influenza outbreaks frequently occur in chicken farms. Currently, most commercially available rapid influenza diagnostic kits can only distinguish between type A and type B influenza viruses. Since the first H9N2 inactivated vaccine was developed in 1988, China has implemented long-term vaccination programs in poultry farms to prevent AIV outbreaks (28, 29). Currently, there are over 20 commercially accessible H9N2 vaccines. Nevertheless, the virus persists in circulating even within vaccinated poultry flocks. Most commercial vaccines in China rely on chicken embryos as the primary platform for virus propagation. However, due to lengthy production cycles, complex manufacturing processes, and high costs (15, 30–32), these vaccines cannot guarantee rapid, large-scale supply during outbreaks. Consequently, the development of novel antibody-based drugs against H9N2 AIV has emerged as a promising research direction (33–35).

Antibodies against H9N2 avian influenza virus play a crucial role in viral surveillance and vaccine-based prevention, yet their applications face several limitations (36–38). Monoclonal antibodies (mAbs), while highly specific, require substantial resources for production and application (39). Particularly for humanized or therapeutic antibodies, high-cost mammalian cell culture systems must be employed to ensure antibody purity and bioactivity. Furthermore, mAbs are extremely sensitive to temperature and pH variations during storage and transportation, demanding stringent environmental stability. The high production costs and cold-chain requirements significantly limit their widespread use in low-resource settings.

Polyclonal antibodies (pAbs), being heterogeneous antibody mixtures with multiple antigen targets, exhibit relatively poor specificity. When used in viral detection kits, they may cause cross-reactivity or false-positive results (40, 41). Production variability due to individual animal differences, immunization duration, and antigen dosage can lead to substantial batch-to-batch variations, compromising experimental reproducibility. Similar to mAbs, pAbs are also sensitive to storage conditions, particularly prone to activity loss or protein degradation during freeze-thaw cycles or long-term storage.

In 1993, Hamers-Casterman et al. discovered that camelid species (including camels, llamas, and alpacas) produced homodimeric heavy-chain antibodies lacking light chains in their serum, in addition to conventional tetrameric antibodies (42, 43). In 1995, Flajnik and colleagues first identified a novel antigen receptor, Immunoglobulin new antigen receptor (IgNAR), in the serum of nurse sharks (*Ginglymostoma cirratum*) (44, 45). This receptor is also a light chain-deficient heavy-chain homodimer, with each chain consisting of one variable domain (V region) and five constant domains (C1-C5). IgNAR differs significantly from conventional antibodies. To date, IgNAR has been identified in nurse sharks (*G. cirratum*), carpet sharks (*Orectolobidae*), and horn sharks (*Heterodontus francisci*) (46–49). The variable domain of IgNAR is designated as VNAR (Variable New Antigen Receptor), which contains only two complementarity-determining regions (CDRs) - CDR1 and CDR3. The diversity of VNAR primarily relies on the CDR3 region (50–52). Leveraging the advantages of high stability, strong specificity, and low immunogenicity, we utilized an *Escherichia coli* (*E. coli*) expression system in this study to develop recombinant VNAR-2SP4H and VNAR-1SP2 proteins that efficiently bind to the HA domain of H9N2 avian influenza virus. These VNARs demonstrated strong viral binding and neutralization capabilities at both *in vitro* and cellular levels. Based on our research findings, these results provide valuable insights for future applications in vaccine development and disease prevention strategies VNAR demonstrates broad application prospects in the diagnosis, detection, and treatment of H9N2 avian influenza virus. Through the integration of genetic engineering and nanotechnology, VNAR holds great potential to advance H9N2-related research and applications to a higher level, offering novel solutions for the prevention, control, and treatment of avian influenza viruses.

## 2 Materials and methods

### 2.1 Immunization and sample collection in *chiloscyllium plagiosum*

Most shark species used for VNAR generation are difficult to maintain in captivity due to their endangered status, large body size, slow maturation, aggressive temperament, or rapid mobility. In contrast, the whitespotted bamboo shark (*Chiloscyllium plagiosum*) is a small, coastal benthic species characterized by docile behavior, hardiness, and early maturation. Through artificial acclimatization, this species can be successfully adapted for laboratory rearing, making it suitable for large-scale aquaculture and subsequent VNAR characterization studies.

For VNAR production, male whitespotted bamboo sharks with body lengths of 50–65 cm were preferentially selected. Upon arrival, sharks underwent a 4-hour acclimatization period to adapt to tank conditions. The artificial seawater shark breeding system comprises a cubic tank with dimensions of 5m×1.2m×1.5m. The density of the seawater is regulated to fall within the range of 1.024 g/cm³ to 1.026 g/cm³ by means of sea salt. The tank is linked to an underlying seawater filtration system, which encompasses a chiller for maintaining the temperature of the artificial seawater at 25°C, a protein skimmer, an algae tank, and a sponge filter. This entire system ensures the fundamental stability of the artificial seawater within the tank. Following gradual introduction into the aquaria, their behavior was closely monitored for signs of stress. Immunization experiments commenced after one week if sharks exhibited normal adaptation. Sharks were fed fresh prawns once weekly. Uneaten food was removed 1 hour post-feeding, followed by partial water replacement to maintain water quality. Full water changes were performed weekly, with concurrent monitoring of chlorine residual, ammonia, nitrite, nitrate, dissolved oxygen, and phosphate levels. Any abnormal parameters were immediately corrected to minimize morbidity/mortality during immunization.

The inactivated H9N2 avian influenza virus served as the immunizing antigen, with the source of the virus strain detailed in Section 2.2. Owing to the requirements of the strain owner and the necessity for patent protection, this study is unable to disclose the specific epitope of the H9N2 virus to which the prepared specific VNAR binds. The antigen was diluted to a concentration of 2 μg/μL using PBS buffer, and each shark was administered an immunization dose of 100 μg in a volume of 100 μL. Prior to immunization, an equal volume of a biphasic adjuvant (SEIWEI 201, Sino-Science Gene) was incorporated into the antigen. Following emulsification, the resultant mixture was subcutaneously injected into the dorsal side of the pelvic fin of each shark, the shark was immunized six times with the same dose of antigen, with a 2-week interval between each immunization. Blood samples were collected from sharks before, during, and after immunization. Prior to blood collection, the shark was comprehensively anesthetized using 500 μL of MS-222. Subsequently, blood was extracted from the caudal vein via a 1 mL syringe (needle length: 13 mm), with approximately 1 mL of blood being collected per instance. The blood was placed in collection tubes and kept at 4°C overnight. Serum was obtained by centrifugation the following day, aliquoted, and stored at -80°C. At the end of immunization, spleen tissues were collected, rinsed twice with PBS buffer, and preserved in cryovials containing RNA stabilization solution (RNALater™, Beyotime). The spleen samples were thoroughly minced into small fragments and then stored at 4°C overnight to ensure complete permeation of the RNA stabilization solution. Subsequently, they were transferred to -80°C for long-term preservation. The serum was employed to assess immunization efficacy via Western blot and hemagglutination inhibition assays, whereas the spleen was utilized for the construction of a primary phage display library.

### 2.2 Evaluation of immunization efficacy in *chiloscyllium plagiosum*

Following the immunization period, qualitative analysis of serum IgNAR was performed by Western blotting. Serum samples were mixed with appropriate protein loading buffer and denatured by heating at 99°C for 10 minutes. After centrifugation at 12,000 rpm for 5 minutes at room temperature, the supernatants were loaded for electrophoresis. Following constant-voltage electrophoresis, proteins were transferred to membranes at 100 V constant voltage. After antibody incubation, the membrane surface was uniformly covered with ECL developing solution, and signals were captured using a chemiluminescence imaging system with subsequent image preservation.

Inactivated H9N2 virus (derived from vaccine strain ZJ01-H9N2/PR8, with HA and NA from A/duck/Zhejiang/01/2021 (H9N2, ZJ01) and six internal genes from vaccine strain A/Puerto Rico/8/34 (PR8, H1N1), inactivated by high temperature (65°C 20min) after chicken embryo culture) was coated at a dose of 1 µg per well. The titer of specific single-domain antibodies IgNAR in immunized shark serum was evaluated using indirect ELISA. After coating, the plates were washed with TBST, and then incubated with different dilutions of immunized shark serum (from 1:100 to 1:100000) at room temperature for 2 hours. After incubation, the plates were washed with TBST, and negative shark serum at the same dilutions was used as a control. After serum incubation and washing with TBST, the plates were incubated with rabbit anti-IgNAR-HRP antibody prepared in this experiment. Then, TMB chromogenic substrate was used to develop color in each test sample. The ELISA plate was placed in a microplate reader (Infinite 200 PRO, Tecan) to measure the absorbance at 450 nm.

Serial dilutions of test serum are mixed with a standardized amount of viral antigen (typically 4 hemagglutinating units), followed by addition of a standardized suspension of RBCs (usually 1% chicken RBCs). After incubation at room temperature for 30–60 minutes, the HI titer is determined as the highest serum dilution that completely inhibits hemagglutination, typically expressed as the log2 value of the reciprocal dilution. This assay is widely used for evaluating influenza vaccine immunogenicity, viral serotyping, and epidemiological surveillance. To ensure accuracy, serum samples require pretreatment with receptor-destroying enzyme (RDE) to remove nonspecific inhibitors, and proper controls including virus back-titration, RBC controls, and positive/negative serum controls must be included.

### 2.3 Phage library construction and panning

The spleen was retrieved from -80°C storage and thawed. The tissue RNA protection solution was discarded, and the spleen was transferred to a clean Eppendorf tube. Then, it was ground with liquid nitrogen to a fine powder. One milliliter of pre-cooled Trizol was added and further ground with a pestle. The mixture stood at room temperature for 5–10 minutes, then was centrifuged at 12000 rpm at 4°C for 10 minutes. The supernatant was transferred to a new Eppendorf tube in a sterile hood. It was mixed with chloroform at a 5:1 ratio, vortexed for 15 seconds, and incubated at room temperature for 3 minutes. After that, it was centrifuged at 12000 rpm for 15 minutes. Three layers (RNA, DNA, and protein from top to bottom) were visible. The supernatant was collected and mixed with an equal volume of isopropanol, vortexed for 15 seconds, and incubated on ice for 15 minutes. Then it was centrifuged for 10 minutes, and the bottom precipitate was collected. The precipitate was washed with 75% ethanol (prepared with DEPC water), and the supernatant was discarded after centrifugation. The tube was inverted in the sterile hood to air-dry. Appropriate DEPC water was added to dissolve the RNA. The concentration and purity were measured, and the RNA was reverse-transcribed (PrimeScript™ III RT-qPCR Mix, TAKARA) into cDNA and stored at -20°C.

VNAR fragments were amplified through nested PCR, while the p3Rdv plasmid vector was separately extracted. Subsequently, the vector and VNAR fragments were digested with restriction enzymes and ligated to construct recombinant plasmids. These recombinant plasmids were then introduced into MC1061F’ electrocompetent cells (Invitrogen) via electroporation (3 KV, 25 μF, 200 Ω, 2 mm) to construct a primary phage display library. And then followed by nested PCR performed using in the first step primers IgNAR-F9~IgNAR-R7, and for the second step primers IgNAR-F2~vNAR_Back (23).

The harvested primary library bacterial culture was amplified and centrifuged, followed by filtration of the supernatant through a 0.22 μm membrane. PEG8000/NaCl was added to the filtrate with thorough mixing, and phage particles were precipitated at 4°C. Phage titers were determined by gradient dilution and plate counting. Three rounds of biopanning were then performed using negative selection (Bovine serum albumin (BSA)-coated plates) and positive selection (H9N2 antigen-coated plates), with overnight incubation at 4°C. After blocking with 3% BSA, phages that bound specifically to H9N2 but not to BSA were eluted and recovered. The eluted phages were used to infect log-phase MC1061F’ cells, and 10 μL of the infected culture was serially diluted for output titer calculation. The remaining culture was supplemented with helper phage and antibiotics for overnight amplification. Subsequent rounds of biopanning (second and third) followed the same procedure with modified conditions as specified in the **Table 1**. Additional rounds of selection could be implemented if necessary to enrich target-specific clones until successful isolation was achieved. Through the measurement of phage titer in each round and the reduction of antigen dose in each round, increasingly rigorous selection conditions can be precisely established. It can be noted that the Output/Input ratio gradually rises with each selection round, suggesting the progressive enrichment of positive clones. In conjunction with the colorimetric results of monoclonal ELISA, this further indicates that specific sequences in the phage library are gradually enriched during the three-round selection process, and there is a high probability that specific VNAR antibody sequences targeting the H9N2 avian influenza virus can be screened out.

**Table 1:** Difference of three-round solid-phase panning.

Round	Concentration of coated antigen	Titer of phage (p.f.u.)	The duration of the positive screening (min)	PBST concentration/number of washes	Type of blocking solution
1SP	5 μg/mL	2×10^12^	60	0.10% PBST/6 times	3% BSA
2SP	2 μg/mL	1/3 1SP phage	45	0.25% PBST/8 times	Casein blocking solution
3SP	1 μg/mL	1/3 2SP phage	30	0.50% PBST/10 times	No protein blocking solution(2%)

Following three rounds of biopanning, phage screening was performed. Positive clones identified from the output plates were individually picked into deep-well plates for overnight culture. The next day, cultures were centrifuged in a plate centrifuge and supernatants were completely removed. Each well was supplemented with TEST lysis buffer and vigorously vortexed, followed by incubation on ice for 1 hour with vortexing every 15 minutes, and subsequent overnight incubation at 4°C. Inactivated H9N2 virus was diluted to 2 μg/mL and 100 μL/well was used to coat four ELISA plates overnight at 4°C. Two parallel ELISA approaches were then conducted: (1) For monoclonal ELISA, bacterial lysates from deep-well plates were diluted 1:1 in PBS as primary antibodies, with anti-FLAG-HRP conjugate serving as secondary antibody; (2) For phage ELISA, four-fold serially diluted phage preparations (library, 1SP, 2SP, and 3SP) were used as primary antibodies with anti-M13-HRP conjugate. Based on monoclonal ELISA results, positive clones were selected for sequencing. The obtained sequences were analyzed through NCBI BLAST to identify those exhibiting characteristic VNAR features.

### 2.4 Structural modeling and epitope validation of VNAR

After obtaining the VNAR sequences, the BLAST tool (Version 2.17.0, 2025) on the NCBI website was employed for structural annotation of the VNAR sequences. Sequences containing complete CDR1, CDR3, FR1, and FR3 regions were selected, and the three-dimensional structure of the VNAR was modeled using SWISS-MODEL (Template library is 7fbk.1.B, by using the method of X-RAY DIFFRACTION 1.90 Å).

### 2.5 VNAR competes with anti-HA monoclonal Ab for binding to H9N2

Inactivated H9N2 virus was incubated with different concentrations of recombinant VNAR, at concentrations of 0 μM, 1 μM (1:10000), and 10 μM (1:1000). After incubation at room temperature for 2 hours, the samples were separated by non-reducing SDS-PAGE. Following electrotransfer according to the method described in Section 2.2, the membrane was incubated with a 1:1000 dilution of mouse anti-HA monoclonal antibody (GT423, Sigma-Aldrich) at room temperature for 2 hours. After washing the membrane with TBST, it was incubated with a goat anti-mouse Ab-HRP secondary antibody. After incubation, the membrane surface was evenly covered with ECL developing solution, and the signal was captured using a chemiluminescence imaging system. The image was then saved.

### 2.6 Expression and purification of the screened VNAR

According to the sequencing results, recombinant VNAR sequences with His tag in N-terminal and C-terminal were cloned into a pET-28a (+) and were transformed into *E. coli* BL21 (DE3) cells. Soluble recombinant VNAR containing His-tags was purified from the cell lysate by Ni-NTA resin. The experimental procedure was referred to our previous study (53).

### 2.7 Indirect immunofluorescence assay

MDCK cells were purchased from Procell Life Science & Technology Co., Ltd (Wuhan, China) and cultured in DMEM medium containing 5% FBS and 1% antibiotics. All cells were maintained at 37°C with 5% CO_2_. For experiments, cells were seeded in 48-well plates at 5×10^5^ cells/well and cultured overnight. After cell attachment, H9N2 avian influenza virus was added. At 48 hours post-infection, indirect immunofluorescence was performed. The medium was removed, and cells were fixed with methanol, blocked with 5% BSA, then sequentially incubated with VNAR, rabbit anti-VNAR, and goat anti-rabbit Alexa Fluor 488 antibodies. Finally, images were captured using a fluorescence microscope (Eclipse TI-U, Nikon), relative fluorescence intensity signals were analyzed using ImageJ software.

### 2.8 Virus neutralization assay

MDCK cells at passage number ≤20 were cultured in DMEM supplemented with 5% fetal bovine serum (FBS) and 1% penicillin-streptomycin. All cells were maintained at 37°C in a 5% CO_2_ humidified incubator. For the experiment, cells were seeded in 96-well plates at a density of 3×10^5^ cells per well and incubated overnight until 90% confluency was achieved.

The target VNAR, an irrelevant VNAR, as well as anti-HA monoclonal antibody (negative control), and oseltamivir (positive control) were serially diluted to working concentrations. A 10 TCID_50_ dose of H9N2 virus was prepared and mixed with each treatment solution, followed by incubation at 37°C for 1 h. The original medium in the 96-well plate was removed, and 100 μL of the virus-treatment mixture was added to each corresponding well. Cells were monitored daily for cytopathic effects (CPE), and results were recorded.

### 2.9 Binding ability and stability assay

To evaluate the binding activity towards the target virus, long-term stability, and thermal stability of VNARs, a sandwich ELISA was employed to compare the binding activities of different VNAR groups to the H9N2 virus. Shark negative serum was utilized as a negative control, and an anti-HA monoclonal antibody was incorporated as a positive control in the binding activity analysis.

To assess thermal stability, the binding activities of VNARs were tested subsequent to incubation at 4°C, 37°C, and 56°C for 1 hour, with shark negative serum serving as a control.

In these experiments, 1 μg of VNAR was coated onto ELISA plates overnight, followed by blocking with 3% BSA. Subsequently, incubation with the H9N2 virus was carried out. After incubation, unbound impurities were removed through TBST washing. Then, two recombinant VNARs (at concentrations ranging from 80 μg/mL to 0.0002 μg/mL), or negative serum and mouse anti-HA monoclonal Ab (diluted at 1:1000) were added. After incubation, unbound impurities were washed away using TBST. Subsequently, rabbit anti-IgNAR V region-HRP antibody (Prepared and stored by our laboratory), rabbit anti-IgNAR C region-HRP antibody (Prepared and stored by our laboratory), and goat anti-mouse HRP antibody were added as secondary antibodies for incubation, respectively. After the incubation with secondary antibodies, TMB substrate was added and left in the dark for 7 minutes. The absorbance of each sample at 450 nm was then measured using an ELISA reader (Infinite 200 PRO, Tecan).

### 2.10 Statistical analysis

All *in vitro* experiments were repeated at least three times. The statistical evaluations of the differences between the means of the experimental groups were performed using Student’s *t*-test, and the data were expressed as the mean ± SD. The criteria for statistical significance used were *p < 0.05, ***p < 0.001 and NS for *p* > 0.05 (GraphPad Prism 8.0). The TCID_50_ value was calculated using the Reed-Muench method formula. The data were analyzed with GraphPad Prism statistical software (version 8.0 for Windows).

## 3 Result

### 3.1 Detection of immune levels in the whitespotted bamboo shark

**Figure 1:**
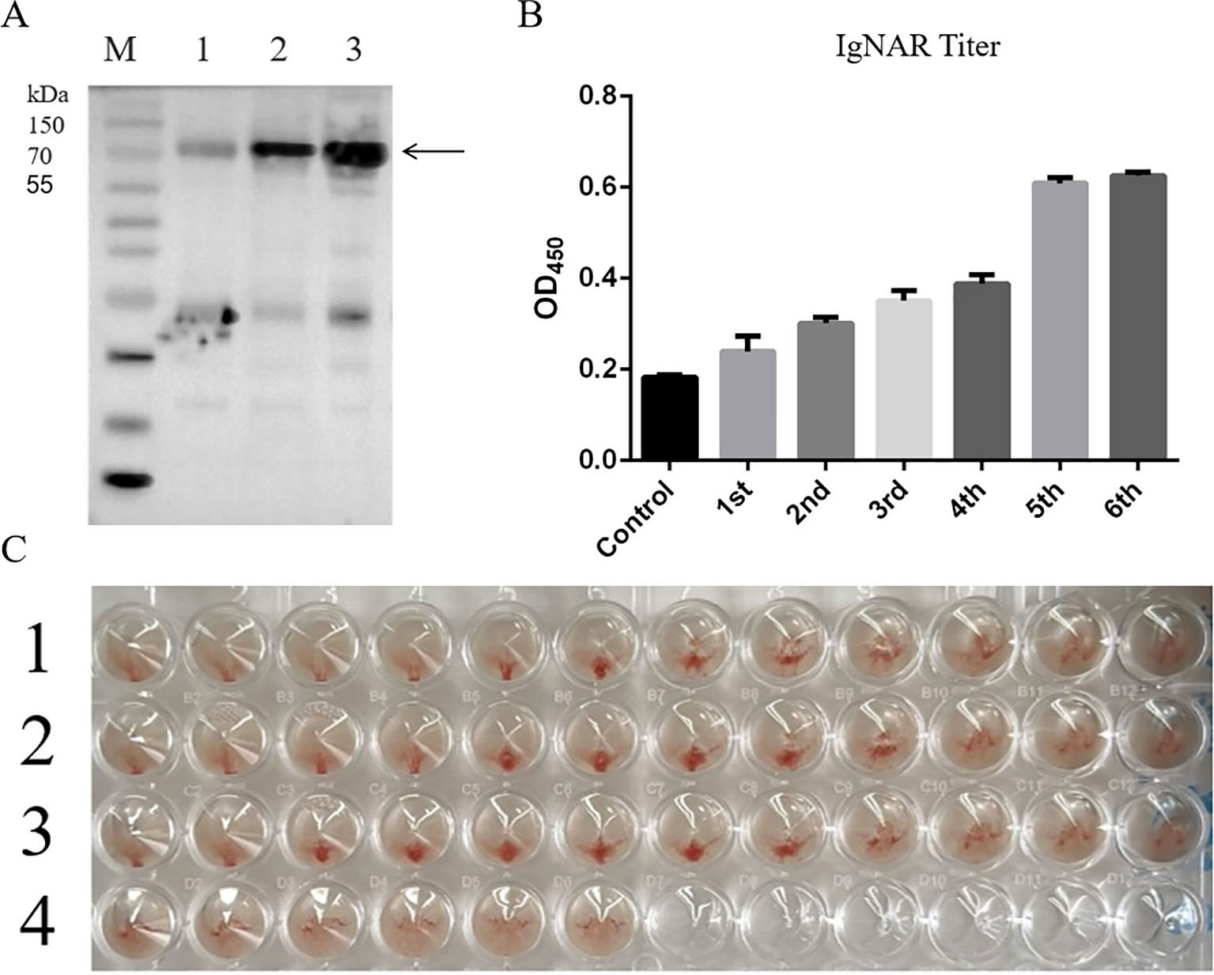
Western Blot test results shark sera from different immunizations. **(A)** M, Protein Marker; 1, The negative serum; 2, The fifth immunization; 3, The sixth immunization (The arrow points to the target band.); **(B)** The results of total IgNAR titer test. Control represents the IgNAR titer in the serum of non-immunized sharks, while the others represent the IgNAR titers in sharks after the first to sixth immunizations; **(C)** Results of shark positive serum hemagglutination inhibition test. 1, Shark seropositive sera; 2, Rabbit seropositive sera; 3, Mixture of seropositive shark sera and rabbit anti-IgNAR antibodies; 4, Negative control.

H9N2 inactivated virus was used as an antigen to immunize whitespotted bamboo sharks for subsequent experiments. Western blot analysis was performed to qualitatively assess seropositive sera from sharks immunized with varying doses (**Figure 1A**). Rabbit anti-*C. plagiosum* IgNAR antibodies (Abbreviated as Rabbit anti-IgNAR Ab, specific binding of IgNAR), purified in our previous studies (53), served as primary antibodies for Western blot detection. As shown in **Figures 1A**, the target band appeared at approximately 90 kDa, consistent with the expected molecular weight of IgNAR. Compared to non-immunized sharks, the IgNAR titer in the serum of sharks immunized with the H9N2 virus antigen gradually increased over six immunization cycles (**Figure 1B**). The result of the ELISA (**Supplementary Figure 1**) further indicated that, in comparison with non-immunized serum, the affinity of shark serum immunized with viral antigen for specific binding to the H9N2 virus was significantly elevated.

Hemagglutination inhibition (HI) assays demonstrated that immunized shark sera generated specific antibodies against H9N2 avian influenza virus, as shown in **Figure 1C**. The results indicated that after six immunization cycles, the shark seropositive sera exhibited higher antibody concentrations and superior viral inhibition compared to immunized rabbit sera. The HI titer of shark sera reached 2^5^, whereas rabbit sera showed an HI titer of 2^4^. When shark sera were co-incubated with rabbit anti-IgNAR antibodies, the mixed solution exhibited reduced viral inhibition, confirming the presence of virus-binding antibodies in shark sera.

### 3.2 Selection outcomes of anti-H9N2 VNAR phage display biopanning

**Figure 2:**
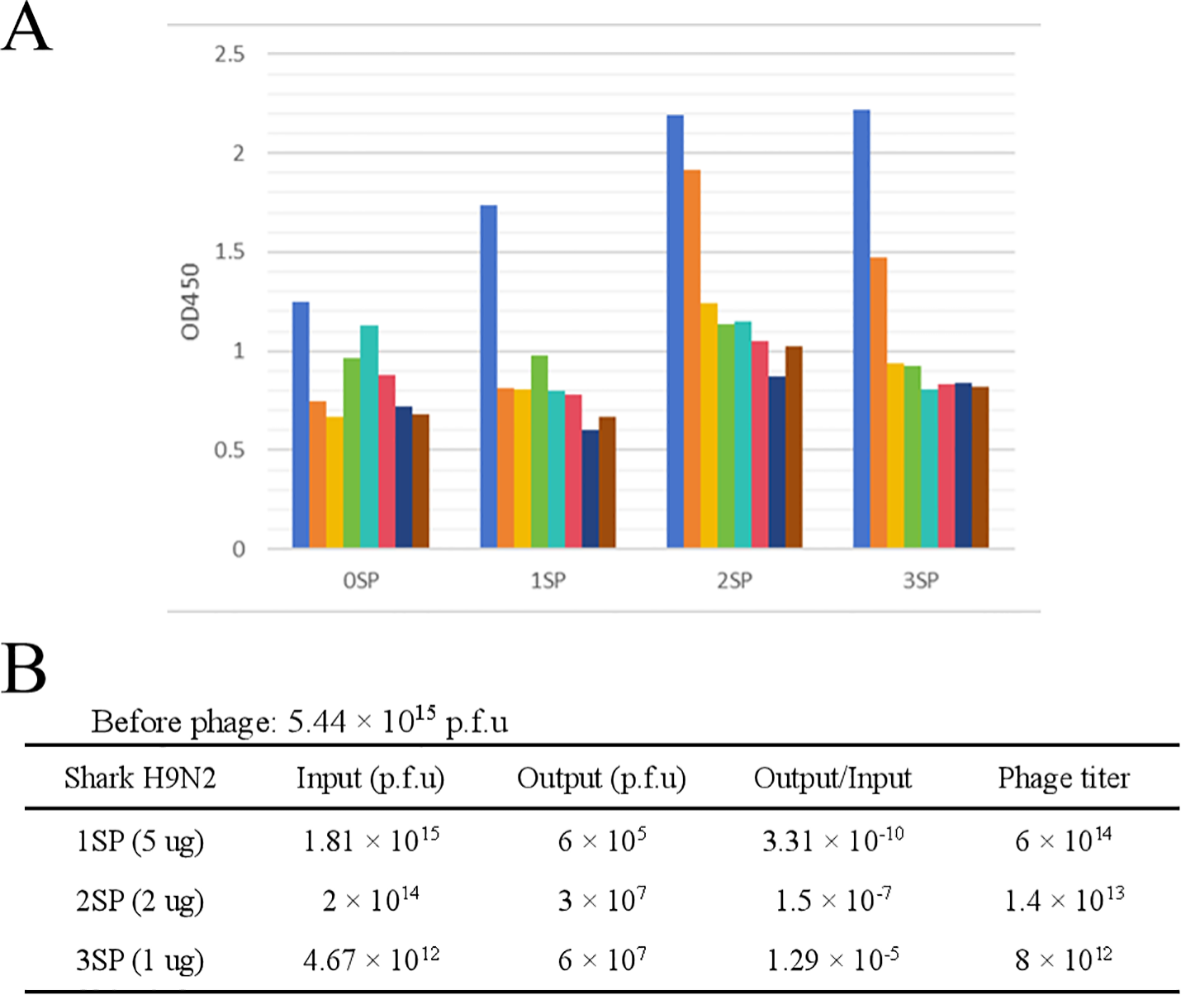
Establishment of the phage display library and results of three-round biopanning enrichment. **(A)** ELISA results from three-rounds of phage display selection. **(B)** Enrichment of target-specific sequences through three-round biopanning.

A primary library with a capacity of 7 × 10^8^ clones was successfully constructed, meeting the requirements for subsequent biopanning experiments (**Supplementary Figure 3**). A large antibody library is critical for obtaining high-affinity binders and ensuring sufficient antibody diversity. Three rounds of phage display panning were performed under increasingly stringent conditions. The outcomes of the monoclonal ELISA conducted throughout the three rounds of selection further illustrate that specific sequences within the phage library are progressively being enriched during the selection procedure. Moreover, there is a high probability that specific VNAR sequences targeting the H9N2 avian influenza virus will be screened out (**Figure 2A**). As shown in **Figures 2B**, the Output/Input ratio progressively increased, indicating effective enrichment of antigen-specific clones. The panning trend confirmed successful selection. Following enrichment, antigen-positive clones were screened via ELISA. Selected clones underwent negative selection to eliminate non-specific binders.

**Figure 3:**
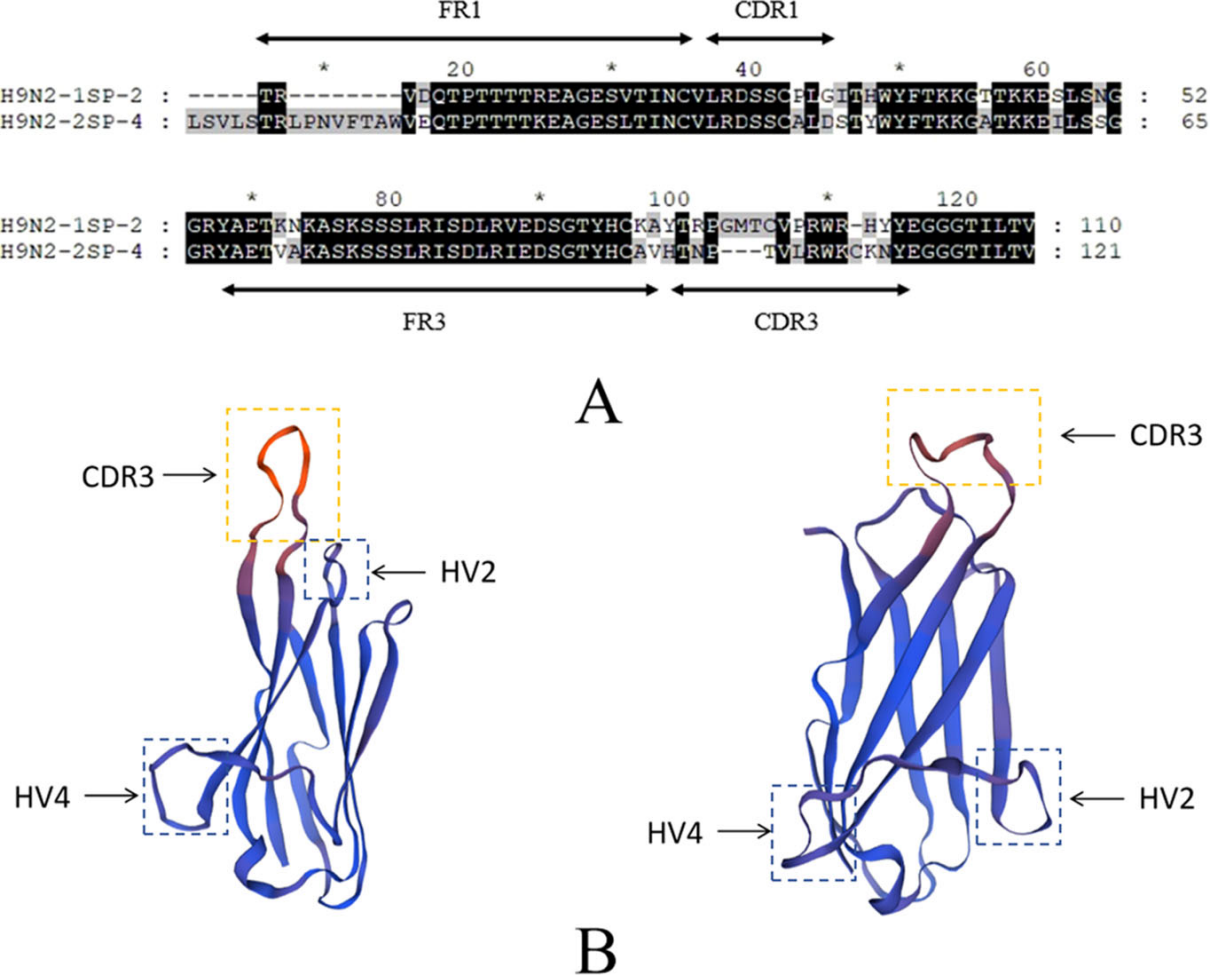
**(A)** Annotation results of the two target sequences. **(B)** Development of VNAR structural prediction model. The arrows point to the main antigen-binding domain within VNARs, Complementarity-Determining Region 3 (CDR3) and Hypervariable region 2 and 4 (HV2 and HV 4).

Through NCBI CD-Search analysis, two sequences containing complete VNAR structural domains were selected for further characterization (**Table 2**). The selected sequences were annotated using GENEDOC software with reference to established literature (**Figure 3A**). Subsequent homology modeling was performed using SWISS-MODEL to generate three-dimensional structural predictions, which demonstrated proper folding into characteristic VNAR frameworks (**Figure 3B**).

**Table 2:** Amino acid sequence.

Name of sequence	Amino acid sequence
H9N2-1SP2G	GSTRVDQTPTTTTREAGESVTINCVLRDSSCPLGITHWYFTKKGTTKKESLSNGGRYAETKNKASKSSSLRISDLRVEDSGTYHCKAYTRPGMTCVPRWRHYYEGGGTILTV
H9N2-2SP4H	GSLSVLSTRLPNVFTAWVEQTPTTTTKEAGESLTINCVLRDSSCALDSTYWYFTKKGATKKEILSSGGRYAETVAKASKSSSLRISDLRIEDSGTYHCAVHTNPTVLRWKCKNYEGGGTILTV

### 3.3 Large-scale expression and purification of anti-H9N2 VNAR

**Figure 4:**
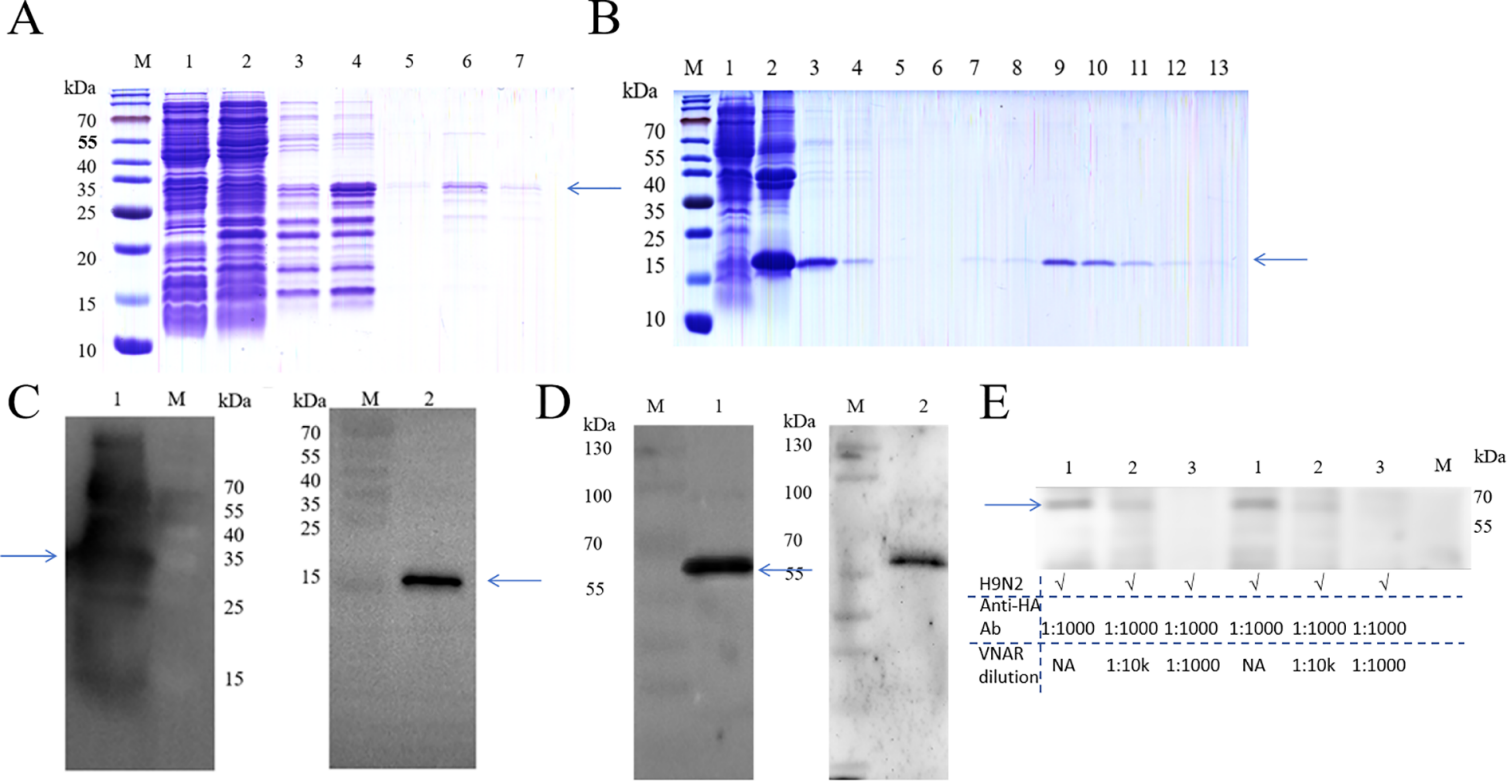
Expression and characterization of recombinant VNARs. **(A)** SDS-PAGE of H9N2-1SP2G purification. Lane M, Protein prestained Marker; Lane 1-7, Flow-through, 10 mM imidazole elution (1st), 10 mM imidazole elution (3rd), 50 mM imidazole elution (1st), 50 mM imidazole elution (5th), 500 mM imidazole elution (1st), 500 mM imidazole elution (5th). **(B)** SDS-PAGE of 28-H9N2-2SP4H purification. Lane M, Protein prestained Marker; Lane 1-13, Lysate supernatant, lysate pellet, refolding solution, flow-through, 10 mM imidazole elution (1st), 10 mM imidazole elution (5th), 50 mM imidazole elution (1st), 50 mM imidazole elution (5th), 500 mM imidazole elution (1st to 5th). **(C)** Western blots of purified recombinant proteins. Lane M, Protein prestained Marker; Lane 1-2, Purified proteins of H9N2-1SP2G, 28-H9N2-2SP4H. **(D)** Western blot analysis of antigen-binding specificity. Lane M, Protein prestained Marker; Lane 1-2, Identification of H9N2 avian influenza virus HA binding site, characterization of 28-H9N2-2SP4H binding specificity to H9N2 subtype influenza virus. **(E)** Specific VNAR (28-H9N2-2SP4H) competitively bind to H9N2 virus with anti-HA monoclonal antibodies by using Western blots. Lane M, Protein prestained Marker; H9N2 virus was incubated with recombinant VNAR at concentrations of 0 μM (Lane 1), 1 μM (1:10000, Lane 2), and 10 μM (1:1000, Lane 3). The arrow points to the target bands.

Since the pET-32a(+) vector contains an N-terminal TrxA solubility tag to facilitate proper protein folding and soluble expression, the TrxA tag can be removed by recombinant enterokinase digestion, yielding correctly folded VNAR. After optimizing the induction conditions, large-scale expression of the two proteins was initiated. However, during small-scale condition screening and large-scale purification, H9N2-2SP4H exhibited significant contamination with host proteins, which could not be effectively removed during column washing to obtain a relatively clear and single target band. Therefore, we decided to perform codon optimization and synthesize the full-length gene, which was then cloned into the pET-28a(+) vector and named 28-H9N2-2SP4H. After column purification, the protein was dialyzed for desalting and concentrated via ultrafiltration, the purification and desalting processes of the two VNARs are shown in **Figures 4A, B**. Since subsequent *in vitro* activity assays were planned, endotoxin removal was performed before protein quantification. Western blotting was then conducted using an anti-His tag antibody as the primary antibody (**Figure 4C**).

After confirming the identity of the purified recombinant proteins, we performed binding site validation of the recombinant VNAR with H9N2 antigen. Since H9N2-1SP2G contained more impurity proteins compared to 28-H9N2-2SP4H after purification, 28-H9N2-2SP4H was selected for antigen-binding site validation (**Figure 4D**). For the H9N2 subtype avian influenza virus, anti-HA antibody was used for incubation, clearly revealing the position and size of the HA band. Comparison with another group incubated with 28-H9N2-2SP4H protein and anti-His antibody demonstrated that 28-H9N2-2SP4H specifically bound to the HA protein of H9N2 subtype avian influenza virus. To further clarify whether the screened recombinant VNAR binds to the HA domain of H9N2, we performed molecular docking predictions of the recombinant VNAR 28-H9N2-2SP4H protein with the H9N2 envelope protein (**Supplementary Figure 2**). Simultaneously, competitive binding experiments were conducted targeting the potential HA binding epitope. The experimental results (**Figure 4E**; **Supplementary Figure 2**) showed that the screened recombinant VNAR can effectively bind to the HA epitope of H9N2. As the concentration of recombinant VNAR co-incubated with H9N2 increased, the commercially available avian influenza virus HA monoclonal antibody gradually lost its ability to bind to the HA epitope of H9N2. This indicates that our screened recombinant VNAR 28-H9N2-2SP4H can effectively bind to the target virus, thus possessing potential virus neutralization capabilities.

### 3.4 Affinity activity and environmental stability assessment of VNAR

The affinity and environmental stability of VNARs were determined by sandwich ELISA. VNARs were serially diluted starting from 80 μg/mL, mixed with 100 μL of antigen, and incubated alongside negative controls containing equivalent concentrations of non-immunized shark serum.

**Figure 5:**
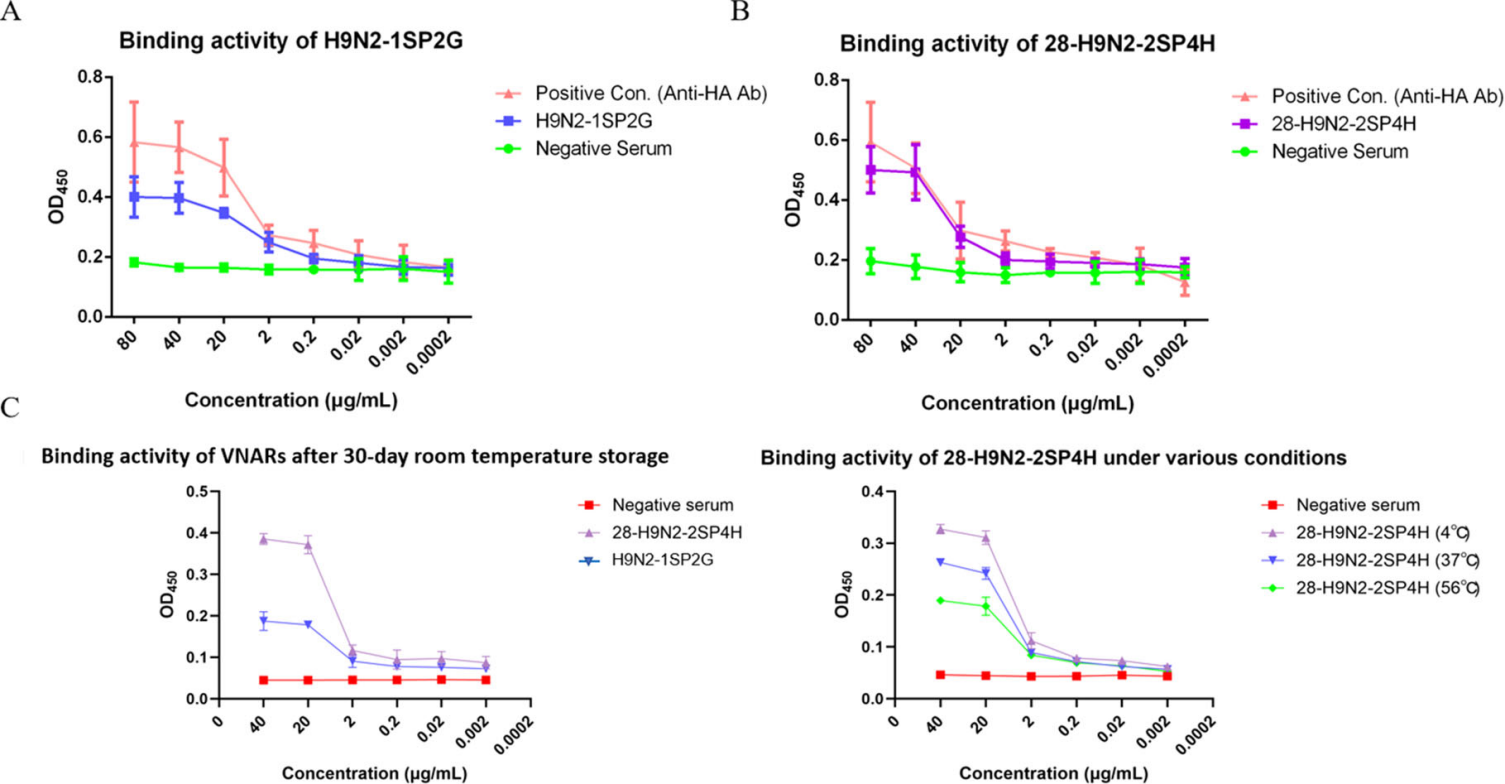
Affinity and environmental stability analysis of recombinant VNARs. **(A)** Binding activity of H9N2-1SP2G to H9N2 virus by sandwich ELISA compared to negative serum and anti-HA antibody. **(B)** Binding activity of 28-H9N2-2SP4H to H9N2 virus by sandwich ELISA compared to negative serum and anti-HA antibody. **(C)** Binding activity of 28-H9N2-2SP4H and H9N2-1SP2G over different storage durations by sandwich ELISA. **(D)** Thermostability of 28-H9N2-2SP4H at varying temperatures by sandwich ELISA.

As depicted in **Figures 5A, B**, both VNARs exhibited enhanced *in vitro* binding activity towards the H9N2 avian influenza virus when compared with the negative control groups. At a protein concentration of 40 μg/mL, the binding activity had essentially reached their maximum. Furthermore, compared to the binding ability of the positive control anti-HA monoclonal Ab, the single-domain Ab 28-H9N2-2SP4H showed stronger binding to the target virus than the other VNAR. The 28-H9N2-2SP4H protein exhibited distinct advantages including single-band purity, high yield, and elimination of enzymatic cleavage requirements, suggesting greater potential for industrial-scale production. **Figure 5C** reveals that while recombinant VNARs retained partial activity after 30 days at room temperature, their binding capacity was reduced compared to samples stored at -80°C post-purification. Thermal stability tests (**Figure 5D**) demonstrated gradual activity loss when recombinant VNARs were exposed to increasing temperatures (4°C, 37°C, and 56°C) for 1-hour intervals. In this experiment, it was observed that the values of the negative control in **Figures 5A, B** were significantly higher than those of the negative serum samples in **Figures 5C, D**. The negative serum samples employed in these experiments were all derived from the same non-immunized shark. Nevertheless, it is plausible that the negative serum samples in **Figures 5C, D** were subjected to different treatments (heat treatment at 56°C or storage at room temperature for one month), resulting in a significant reduction in their absorbance values during the ELISA assay. However, this phenomenon necessitates further exploration.

### 3.5 Blocking activity of recombinant VNAR at the cellular level

**Figure 6:**
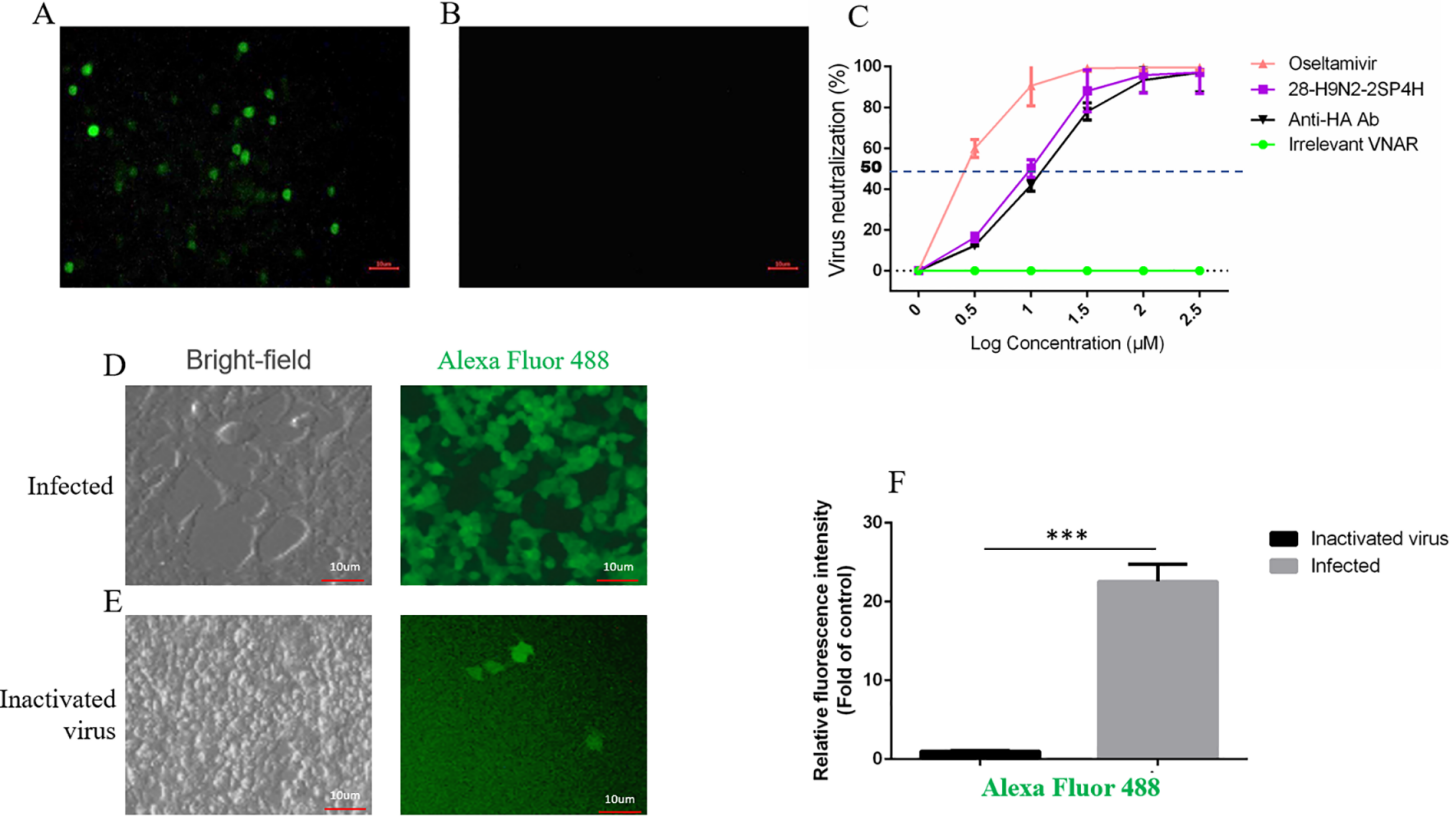
Evaluation of recombinant VNAR binding blockade activity at cellular level. **(A)** Indirect immunofluorescence results of recombinant VNAR and H9N2 subtype avian influenza virus (100 ×10μm). **(B)** Negative control (100 ×10μm). **(C)** Neutralization of H9N2 virus by different concentrations of VNAR compared to irrelevant VNAR, anti-HA monoclonal antibody and Oseltamivir. Infected cells **(D)** and mock infected cells **(E)** were detected using bright-field and fluorescence microscopy. **(F)** Relative quantification of fluorescence signal intensity (Alexa Fluor 488) in MDCK cells treated with H9N2 or inactivated H9N2 virus, n = 3, *** for *P* value <0.001.

Evaluation of recombinant VNAR blocking activity at cellular level by indirect immunofluorescence and antibody neutralization assays. The cellular immunofluorescence results are shown in **Figures 6A, B**. When cells were infected with H9N2 subtype avian influenza virus, the purified recombinant VNAR successfully bound to the virus. Following incubation with our laboratory-produced rabbit anti-VNAR antibody, Alexa Fluor 488 conjugated to the rabbit antibody and generated green fluorescence signals, while the irrelevant VNAR incubated cells showed no fluorescence signal, demonstrating that the recombinant VNAR specifically recognized and bound to H9N2 subtype avian influenza virus on the cells. Analysis of the state of MDCK cells after infection (**Figures 6D, E**) showed that the H9N2 virus proliferated extensively within the cells, exhibiting a strong fluorescent signal after VNAR binding, and causing significant cell death and typical cytopathic effects (CPE). In contrast, cells treated with inactivated H9N2 virus maintained their structural integrity. After washing the cells with PBS and adding VNAR, there was virtually no fluorescent signal (**Figure 6F**), indicating that the recombinant VNAR we prepared possesses strong binding specificity to the H9N2 virus.

In the TCID50 assay of the H9N2 virus (**Table 3**), we diluted the P1 generation virus from 10–^1^ to 10^-8^. We then counted the number and proportion of target cells showing cytopathic effects (CPE) at each dilution (**Table 3**). According to the Reed-Muench method, the TCID50 of the P1 generation H9N2 virus was calculated to be 10^-2.9^/0.1 mL. This means that diluting this batch of virus 10^2.9^ times and inoculating 100 μL will cause 50% of the cells to show cytopathic effects.

**Table 3:** Results of P1 generation H9N2 avian influenza virus TCID_50_.

Virus dilution	Number of cells showing CPE	Number of cells with no CPE	Cumulative number of cells (showing CPE)	Cumulative cell count (no CPE observed)	Total number of cell wells	Cells well showing CPE/%
10^-1^	8	0	20	0	20	100
10^-2^	6	2	12	2	14	85.7
10^-3^	3	5	6	7	13	46.2
10^-4^	2	6	3	13	16	18.8
10^-5^	1	7	1	20	21	4.8
10^-6^	0	8	0	28	28	0
10^-7^	0	8	0	36	36	0
10^-8^	0	8	0	44	44	0

The neutralization assay was performed after determining the TCID_50_ value of H9N2 virus. As shown in **Figures 6C**, the experiment included three groups: antibody (VNAR) dilution series, irrelevant control, and positive control (Oseltamivir) groups. The antibody dilution group demonstrated a dose-dependent inhibitory effect on the virus, where the inhibition rates were positively correlated with the antibody concentration (10 μM of the screened VNAR was capable of protecting 50% of the virus-targeted cells from CPE, with an EC_50_ value of 10.0 μM, meanwhile, the Oseltamivir treatment resulted in a virus neutralization EC_50_ of 1.7 μM). In the irrelevant control group, all cells were infected by the H9N2 virus, commercially available anti-HA monoclonal antibody showed limited neutralizing ability against the virus, with an EC_50_ value of 16.1 μM, indicating that it is less effective at protecting MDCK cells from infection compared to the VNAR we screened. In contrast, in the positive control group, when treated with Oseltamivir at a concentration starting from 1 µM, the cells remained highly uninfected and showed favorable survival rates.

## 4 Discussion

The surface antigens of viral particles predominantly comprise HA and NA proteins, with HA acting as the principal target for antibody-mediated therapeutic efficacy. Antibodies directed against HA can be generally classified into two categories: those targeting the HA head region and broadly neutralizing antibodies (54–56). Head-targeting antibodies primarily recognize the receptor-binding site (RBS) or adjacent regions of the HA head domain, whereas broadly neutralizing antibodies mainly target conserved regions in the HA stem, such as the cross-subtype conserved fusion peptide or helical stalk regions (57).

Although this study has successfully identified VNAR antibodies that target the H9N2 viral HA protein and demonstrated their antiviral potential, several questions still await further investigation. The H9N2 subtype displays mutational variability, where the HA stem domain is relatively conserved, whereas the receptor-binding domain (RBD) in the head region experiences frequent mutations. While the study confirmed that recombinant VNARs can effectively bind to the H9N2 HA protein and neutralize the virus, the exact binding epitope could not be verified via sequence alignment or structural modeling because of the confidentiality of the immunizing inactivated virus strain. These results imply that the target may probably be located in conserved regions. The whole-virus immunization strategy employed in this study, in conjunction with phage display technology, generated HA-targeting VNARs. Although, due to the high homology of positive sequences and the lack of critical functional domains in certain sequences, only two VNARs with potential HA-targeting binding activity were ultimately selected, this still indicates that these epitopes possess an immunological superiority during natural infection, suggesting that these epitopes display immunodominance in natural infections. Functional constraints on HA may preserve certain conserved regions under evolutionary pressure, rendering such epitopes inherently resistant to mutations. Presently, there are no reported investigations on VNAR targeting HA. By assessing the core technological merits of shark single-domain antibodies (VNARs), such as their outstanding deep epitope recognition capacities, high stability, and diverse applications, it is hypothesized that VNARs may exhibit broad neutralizing capabilities against a variety of influenza viruses, including influenza B virus (IBV) and influenza A virus (IAV) (58, 59). Notably, the recombinant VNARs maintained the same specific binding activity against both high-temperature inactivated and normal H9N2 viruses, this result suggests that it still possesses potential effective neutralizing activity against avian influenza virus strains different from the immunizing strain, further supporting the hypothesis of conserved region targeting (58). Future studies could evaluate epitope conservation through cross-neutralization tests with historical strains, engineer existing VNARs to target emerging variants, or develop multivalent neutralizing systems combining antibodies against different HA domains to reduce the risk of single-epitope escape mutations. Currently, only a limited number of reported studies have concentrated on single-domain antibodies targeting avian influenza viruses. In our research on anti-H9N2 virus agents, we selected shark-derived VNARs rather than camelid-derived VHHs. The primary reason is that the procurement cost of *Chiloscyllium plagiosum* is less than one percent of that of the alpaca, a typical animal model for VHH production. This enables the large-scale development and production of VNARs. Moreover, recombinant VNARs expressed in *E. coli* prokaryotic expression system display high activity, providing substantial advantages in subsequent VNAR production and genetic modification (60). Therefore, it is considered that VNARs derived from *Chiloscyllium plagiosum* are ideal molecules for the development of novel single-domain antibodies. However, in comparison with VHH development, VNAR screening lacks highly efficient primary library cloning primers, and the negative selection steps in the panning process need to be gradually optimized, which may extend the development cycle. Additionally, since sharks are lower vertebrates, their adaptive immune response is more primitive than that of camelids that produce VHHs, and the maturation rate of specific antibodies is slower (61). Humanization of these antibody molecules also encounters difficulties, which is a drawback compared to natural nanobodies such as VHHs.

This research achieved the successful production of the screened recombinant VNAR antibodies specific to the H9N2 HA protein via a prokaryotic expression system. It also verified their robust virus-neutralizing ability, thereby presenting a novel strategy for the control of avian influenza.

Through cell level detection and virus neutralization experiments, it was found that the screened recombinant anti-H9N2 VNAR could effectively bind and protect 50% of virus target cells from CPE at a concentration of 10.0 μM (While the EC_50_ value of the anti-HA monoclonal antibody was 16.1 μM). It showed the potential role of single domain antibody in inhibiting avian influenza virus. The recombinant VNAR prepared in this study exhibited highly specific binding to MDCK cells infected with the H9N2 virus at the cellular level. At relatively high concentrations, the recombinant VNAR displayed comparable viral neutralization capacity and target-cell protection rates to the positive control drug Oseltamivir (**Figure 6**), indicating its antiviral activity. Presently, the prevention and treatment of avian influenza primarily concentrate on the utilization of small-molecule antiviral drugs such as Oseltamivir, Zanamivir, and Baloxavir, with a lack of application of effective antibody drugs (62). Single-domain antibodies targeting the influenza virus hemagglutinin protein may emerge as a frontier area in the current development of anti-influenza drugs and vaccines. Single-domain antibodies possess a relatively small molecular weight (approximately 15 kDa) and a long CDR3 loop, enabling them to penetrate into the grooves of the HA protein that are inaccessible to traditional IgG antibodies (such as the HA stem or the narrow crevices near the receptor-binding site), thereby demonstrating outstanding deep-epitope recognition capabilities (63). Moreover, owing to the unique characteristics of the shark’s bodily fluid environment, VNAR is therefore considered to possess high chemical stability (64). Due to their smaller epitope footprint, single-domain antibodies may exhibit more persistent binding capabilities than traditional monoclonal antibodies when confronted with variants that generate immune-escape mutations (58, 59). These core technological advantages endow single-domain antibodies with broader application potential.

The outcomes of the environmental stability experiment indicated that the recombinant anti-H9N2 VNAR still maintained substantial viral binding activity following long-term storage at 37°C and exposure to a high temperature of 56°C. This finding will significantly broaden the applicable scope for screening anti-avian influenza virus VNARs and establish a foundation for the extensive application of this antibody preparation.

Future research will concentrate on accurately mapping the binding epitopes and assessing *in vivo* biological activity, especially exploring oral bioavailability. Characterized by low production costs, scalability, and notable stability under extreme temperature and pH conditions, VNARs can considerably reduce storage and transportation costs. These characteristics render them promising candidates for the development of colloidal gold rapid diagnostic tests, which are particularly valuable for resource-constrained regions. The utilization of shark-derived targeted VNARs has the potential to promote equitable access to avian influenza diagnosis and prevention measures.

## Data Availability

The original contributions presented in the study are included in the article/**Supplementary Material**. Further inquiries can be directed to the corresponding authors.
