# Supplementary material for: Development and application of a VNAR-based detection nanobody for avian influenza virus H9N2
Source: Front Immunol. 2026 Mar 24;17:1735255. doi: 10.3389/fimmu.2026.1735255 (PMC13053647; doi:10.3389/fimmu.2026.1735255)
Supplement: Supplementary file 1 [file DataSheet1.pdf]

Development and Application of a VNAR-based Detection Nanobody for Avian Influenza Virus H9N2

Hongzhou Ye[1,3], Qingqing Dong[2,3], Min Qian[2,3], Yanwen Guo[2,3], Yi Chen[1], Wenyuan Liu[1], Lili Liu[2], Zhengbing Lyu[2*], Chen Yuan[1*]. Xiaofeng Jiang[2*],

1 Department of Pediatrics, The First Affiliated Hospital of Huzhou University, 158 Guangchanghou Road, Huzhou, 313000, China.

2 College of Life Sciences and Medicine, Zhejiang Sci-Tech University. Zhejiang provincial key Laboratory of Silkworm Bioreactor and Biomedicine, Zhejiang Sci-Tech University, Hangzhou, 310018, China.

3 These authors contribute equally to this work.

1. Immunized shark serum titer testing

After six immunizations with the H9N2 virus, blood was drawn from the caudal vein of the shark. The blood was centrifuged at 4°C to obtain serum, and the serum titer was detected using indirect ELISA. Each well was coated with 1 μg of viral antigen, followed by incubation with serum at different dilution ratios. Non-immunized serum at the same dilution ratios was used as a negative control. After serum incubation, a rabbit anti-IgNAR antibody prepared in our laboratory was used for secondary antibody incubation. The results (Fig. 1B) showed that the positive serum diluted 50,000 times still exhibited strong antigen-specific binding ability, indicating that after six immunizations, the striped bamboo shark exhibits a robust immune response and produces a high abundance of single-domain antibodies against the H9N2 virus..

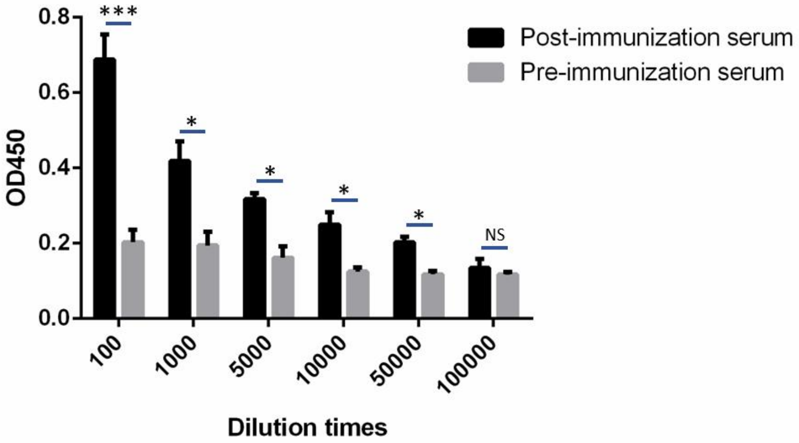

Figure S1. The results of IgNAR titer test. The horizontal axis represents the serum dilution times, and the vertical axis represents the absorbance value.* for *P* value <0.05, *** for *P* value <0.001, NS for not significant.

2. Protein docking prediction analysis of recombinant VNAR and H9N2

To understand the binding ability of the screened recombinant VNAR to the H9N2 viral protein, high-quality 3D structures were selected for protein-protein docking with the H9N2 viral protein. The binding free energy of the protein-protein complex was evaluated using MM/GBSA.

The binding energy of recombinant VNAR (28-H9N2-2SP4H) was -30.82 kcal/mol, and the docking results of the antigen-binding domain CDR3 were favorable, the VNAR protein (28-H9N2-2SP4H) engages in interactions with two sites on the H9N2 HA antigen epitope, specifically ARG-105 and GLY-286, via its LYS-110, GLN-112, and TRP-95 residues. TRP-95 is situated in the Ig strand F domain external to the CDR3 region, whereas LYS-110 and GLN-112 are positioned within the CDR3 region of the VNAR (Fig. S2), indicating its potential for stable binding to the target virus.

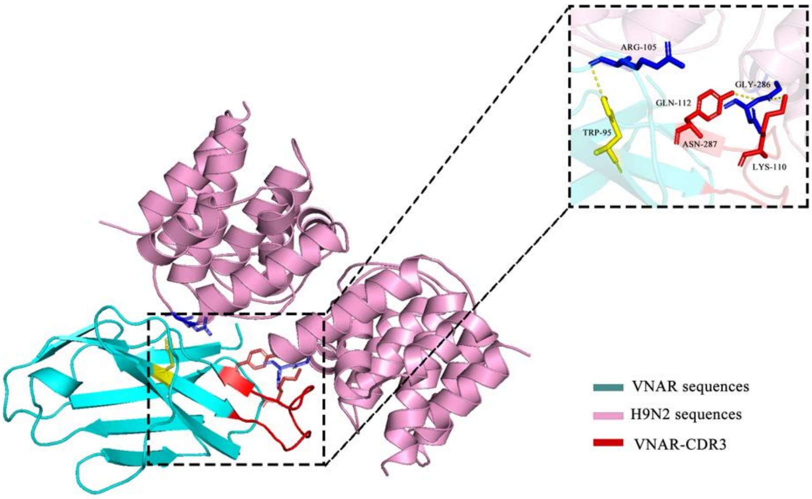

Figure S2. Protein-protein molecular docking prediction result.

3. Bacteriophage library construction results

The results of the bacteriophage library construction are shown in Figure S3. Even at a 10^{-6} dilution, 35 single clones were still observed on the culture plate. The calculated library size is 7×10^{8}, which is sufficient to support subsequent rounds of bacteriophage selection experiments.

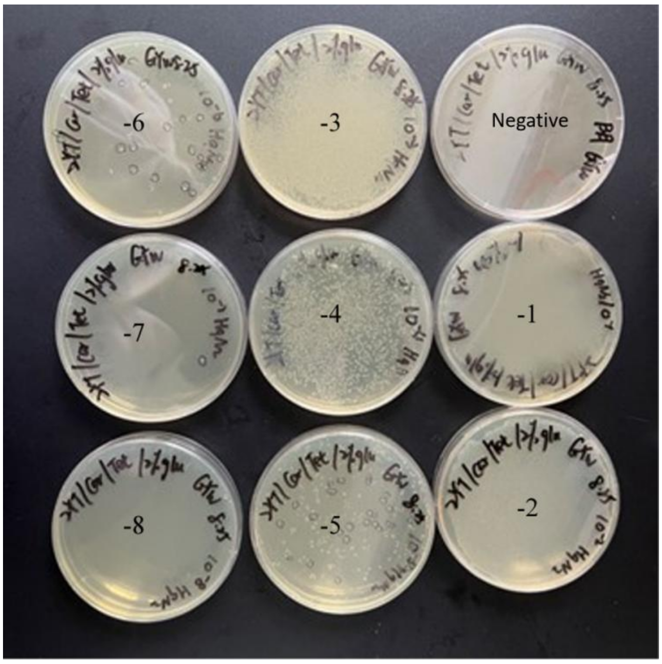

Figure S3. Phage reservoir capacity display.
